# Neutrophils extracellular traps formation may serve as a biomarker for disease activity in oligoarticular juvenile idiopathic arthritis: a pilot study

**DOI:** 10.1186/s13075-023-03104-9

**Published:** 2023-07-31

**Authors:** Merav Heshin-Bekenstein, Szilvia Baron, Grant Schulert, Anna Shusterman, Victoria Fidel, Yoav Ben-Shahar, Rachel Shukrun, Yoav Binenbaum, Ronit Elhasid

**Affiliations:** 1 Pediatric Rheumatology Service, Dana Dwek Children’s Hospital of Tel Aviv Medical Center, Tel Aviv, Israel; 2 School of Medicine, Tel Aviv University, Tel Aviv, Israel; 3 Pediatric Hemato-Oncology Research Laboratory, Tel Aviv Medical Center, Tel Aviv, Israel; 4 Division of Rheumatology, Cincinnati, OH USA; 5 Department of Pediatrics, University of Cincinnati College of Medicine, Cincinnati, OH USA; 6 Department of Pediatric Surgery, Tel Aviv Medical Center, Tel Aviv, Israel; 7 Department of Pediatric Hemato-Oncology, Tel Aviv Medical Center, Tel Aviv, Israel

**Keywords:** Juvenile idiopathic arthritis (JIA), Neutrophil, Neutrophil extracellular traps (NETs), Neutrophil elastase (NE), Myeloperoxidase (MPO)

## Abstract

**Background:**

Juvenile idiopathic arthritis (JIA) is the most common chronic rheumatic disease in children, causing significant morbidity. Despite the dramatic improvement in treatment, many patients do not achieve complete remission, and biomarkers for subclinical disease, flares, and response to treatment are lacking. Neutrophils and neutrophil extracellular traps (NETs) play key roles in the pathogenesis of autoimmune and inflammatory conditions. In this study, we characterized neutrophil enzyme activity and NETs formation in oligoarticular and polyarticular JIA and explored their association with disease activity.

**Methods:**

Neutrophils from 6 healthy controls and 7 patients with oligoarticular and polyarticular JIA were freshly isolated at time of diagnosis and after glucocorticoid intra-articular injection. Enzymatic activity of neutrophil granular enzymes was monitored by colorimetry and PMA-activated NETs formation was assessed using fluorescent microscopy.

**Results:**

In this pilot and feasibility study, we revealed that NETs were significantly increased in oligoarticular JIA patients at time of diagnosis compared to healthy controls. Anti-inflammatory treatment using intra-articular steroid injection normalized NETs formation in these patients. Correlation between NETs formation and clinical Juvenile Activity Disease Activity Score-10 (cJADAS-10) was linear and significant (*P* = 0.007) in oligo but not in poly JIA patients.

**Conclusions:**

This is the first study exploring the link of NETs formation with oligo and poly JIA activity. We demonstrated a statistically significant linear correlation between cJADAS-10 and NETs formation in oligo but not in poly JIA patients. Hence, we suggest that NETs may reflect clinical disease activity in JIA, and may serve as a putative biomarker. Further work is needed to validate these initial results and determine the dynamics of NETs formation in JIA.

## Background

Juvenile idiopathic arthritis (JIA) is the most common chronic rheumatic disease in children and can cause significant morbidity, including joint damage and impaired growth [1]. JIA is categorized into distinct subclasses [2], with oligoarticular (oligo) JIA and polyarticular (poly) JIA being the most prevalent [3–5]_._ Apart from the number of inflamed joints during the first 6 months of disease, some mechanistic and immunologic overlaps exist between these two JIA subtypes. Consistently, there is growing consensus that among positive Anti-Nuclear Antibody (ANA) patients, oligo JIA and rheumatoid factor (RF)-negative poly JIA share many clinical characteristics and may represent a continuum of a single disease entity rather than distinct diseases [6, 7]. Despite the dramatic improvement in the disease treatment by the inclusion of biological therapy, many patients still do not achieve complete remission. Furthermore, biomarkers are needed to improve the goal of “treat to target” in JIA. These markers can assist in the detection of subclinical disease, response to therapy, and prediction of flares.

Neutrophils, the most abundant leukocyte in the circulating blood, represent the first line of defense within the innate immune system [8]. Neutrophils protect the host by several mechanisms including phagocytosis, the release of cytotoxic molecules, and the formation of neutrophil extracellular traps (NETs) [9]. NETs are extruded by activated neutrophils and are composed of DNA fibers, histones, and antimicrobial proteins such as neutrophil elastase (NE) and myeloperoxidase (MPO). NETs trap extracellular pathogens and hence protect organisms from infections [10, 11]. However, in recent years it was demonstrated that NETs are also formed in non-infectious conditions [12] including inflammatory and autoimmune diseases such as systemic lupus erythematosus (SLE) [13–15], rheumatoid arthritis (RA) [16, 17], anti-neutrophil cytoplasmic antibodies-associated vasculitis [18, 19] and inflammatory bowel disease (IBD) [20]. NETs formation has been intensively studied in adult rheumatic diseases and has been proposed to be involved in both its initiation and progression. In RA, increased NETs formation was shown to be associated with increased disease activity and is therefore considered a new biomarker for monitoring disease activity, possibly allowing for early preventive treatment intervention [17]. The role of NETs in pediatric-onset rheumatic diseases in general and in JIA, in particular, has not been adequately addressed [21–23]. Studies in JIA showed a correlation between disease activity and S100A8/A9 proteins, which are primarily present in the cytoplasm of neutrophils and monocytes [24]. S100A12, a marker for neutrophil activation, was also found to be present in the serum and synovial fluid (SF) of JIA patients [23].

Recent study exploring the presence of NET-associated markers in JIA has found that the number of citrullinated histone H3 + neutrophils was higher in the peripheral blood of patients with JIA than in healthy donors, implying an increased NETs formation in JIA. Additionally, they also observed elevated levels of NET-associated products including NE, LL37 (cathelicidin antimicrobial peptide), and cell-free DNA-histone complexes in the same JIA cohort [25].

Understanding the role of neutrophils in JIA can potentially facilitate the detection of subclinical disease and the prediction of disease course. In this study, we aimed to characterize neutrophil enzymes’ activity and NETs formation in oligoarticular and polyarticular JIA and specifically explore its association with JIA disease activity.

## Methods

### Study population

This is a single-center prospective study conducted at Dana Children’s Hospital of Tel Aviv Medical Center, Tel Aviv, Israel. All parents signed an informed consent form in accordance with the Declaration of Helsinki, Institutional Review Board, Tel Aviv Sourasky Medical Center, Tel Aviv, Israel 0502–19-TLV.

The source for this cohort is a larger cohort of 22 children with JIA aged 1–18 years, recruited in the pediatric rheumatology clinic of Dana Dwek Children’s Hospital between 2/2020 and 2/2021. Inclusion criteria for the larger cohort were a diagnosis of JIA by the International League of Associations for Rheumatology criteria (ILAR) criteria [2], with longitudinal data collection including clinical data and blood samples.

For this study, we included only 7 children from the larger cohort, who were those with a diagnosis of oligo- or RF − poly-JIA who had samples from both time of diagnosis and from the time of first follow-up visit after the initiation of the anti-inflammatory treatment, that included intra-articular steroid injection for all patients, and methotrexate initiated at time of the steroid injection in most of the poly-JIA patients. Patients with systemic-onset JIA and RF + poly JIA were excluded from the analysis, as well as patients who were already being treated with anti-inflammatory medications at the time of the recruitment, including NSAIDS and steroids.

In addition, blood samples were obtained from 6 healthy volunteers, aged 1–18, who were hospitalized for elective surgery and were used as controls. These participants had no chronic or inter-current inflammatory disease and did not take any medications.

For JIA patients, all study visits included physical examination for joint count (0–10 active joints), physician’s global assessment (PhGA) of disease activity on a visual analog scale (VAS) (0–10 cm), and parent/patient-reported global assessment (PGA) of well-being, assessed by a parent if the child was ≤ 9 years old (VAS 0–10 cm). A blood sample was drawn on each visit and included white blood cells (WBC), absolute neutrophilic count (ANC), absolute lymphocytic count (ALC), and C-reactive protein (CRP). Antibody profile from the time of diagnosis included anti-nuclear antibody detected by indirect immunofluorescence assay (ANA-IFA), RF, and anti-cyclic citrullinated peptide (anti-CCP). The clinical JADAS-10 (cJADAS-10) was calculated from the sum of the number of active joints, PhGA, and PGA. The Juvenile Activity Disease Activity Score-10 (JADAS-10) was calculated from the sum of the active joint count, PhGA, PGA, and normalized CRP to a scale of 0–10 [26]. In addition to routine laboratory testing, a blood sample was collected for neutrophil isolation to monitor NE and MPO activity, as well as the percentage of NETs forming neutrophils following PMA activation. Correlation between the disease activity score cJADAS10 or NETs formation and the following metrics were assessed: NE activity, MPO activity, and NETs formation.

### Materials

Phosphate buffered saline (PBS) was obtained from Biological Industries. Ethylenediamine tetra-acetic acid (EDTA), bovine serum albumin (BSA), glucose, phorbol 12-myristate 13-acetate (PMA), and Triton X-100 were all purchased from Sigma-Aldrich (MO, USA). Poly-L-lysine solution (0.01%) and buffered 4% paraformaldehyde solution were acquired from Merck (NJ, USA).

### Whole blood collection and neutrophil isolation

Human peripheral blood samples (2–5 mL) in EDTA-coated vacutainer tubes (Greiner Bio-One, Austria) were obtained from all participants at each time point. Fresh blood was processed within 60 min of withdrawal. Neutrophils were isolated using EasySep Direct Human Neutrophil isolation kit (StemCell Technologies Inc., Canada), based on immunomagnetic negative selection, according to the manufacturer’s instructions. The number of isolated neutrophils was quantified using Beckman coulter DxH800 (Beckman Coulter Inc., CA, USA), and the final concentration was adjusted to 10^7^/ml in RPMI.

### Neutrophil elastase enzymatic activity

10^5^ neutrophils were lysed in 0.2% Triton X-100 solution and incubated with 500 µM chromogenic peptide elastase substrate (Calbiochem, CA, USA) for 90 min at 37 °C. Enzymatic activity was measured by an iMark Microplate Absorbance Reader (Bio-Rad, CA, USA) at 415 nm. A calibration curve was set up by using various amounts of purified NE (Athens Research & Technology, GA, USA). Purified NE was used as a positive control, and a specific NE inhibitor IV (Calbiochem, CA, USA) together with the purified enzyme were used as a negative control for each experiment. NE activity was calculated for 10^6^ neutrophils.

### Myeloperoxidase enzymatic activity

10^5^ neutrophils were lysed in 0.2% Triton X-100 solution and incubated with 0.1 mg/ml O-phenylenediamine (Sigma-Aldrich, MO, USA) and 1 mM H_2_O_2_ (Sigma-Aldrich) for 20 min at room temperature. Enzymatic activity was measured by an iMark Microplate Absorbance Reader (Bio-Rad, CA, USA) at 450 nm. A calibration curve was set up by using various amounts of purified MPO (Athens Research & Technology, GA, USA). Purified MPO was used as a positive control, and 4-aminobenzoic acid hydrazide (Cayman Chemicals, MI, USA), a specific MPO inhibitor, together with the purified enzyme were used as a negative control for each experiment. MPO activity was calculated for 10^6^ neutrophils.

### Neutrophil activation and immunofluorescent staining

Neutrophils were seeded on coverslips coated with poly-L-lysine and activated by 100 nM PMA for 3 h at 37 °C. Subsequently, cells were fixed using a 4% paraformaldehyde solution. Neutrophils were labeled with Sytox Green (Thermo Fischer Scientific, MA, USA) and Hoechst 33,342 (Sigma-Aldrich, MO, USA). Imaging was performed on an LSM700 Laser Scanning Confocal Fluorescence microscope (Zeiss). In each sample, 3 regions of interest containing 100–200 cells were evaluated for NETs formation manually. Non-NETting neutrophils were defined as those with compact DNA stained with both nuclear dyes. NETing neutrophils were defined as those having diffuse DNA stained only with Sytox green.

### Statistical analysis

GraphPad Prism version 5 (GraphPad Software Inc., CA, USA) was used for statistical analysis. Student *t*-test or one-way ANOVA test with Bonferroni post hoc test was used to calculate differences. Statistical tests for comparison were two-tailed, and *P < 0.05 was considered significant. The data are presented as mean ± standard error of mean.

## Results

### Patients demographics and clinical characteristics

Seven patients who fulfilled the inclusion criteria were included in the analysis, with a median age of 3 years (range 2–14) and 71% female. 57% of the patients were diagnosed with oligo JIA (*n* = 4) and 43% of the patients with poly JIA (*n* = 3, Table 1). All oligo JIA patients in the cohort were females, with a median age of 4.5 years old (range 3–11). The poly JIA group included 2 males and 1 female, with a median age of 3 years old (range 2–14). In addition, a total of 6 healthy controls were recruited, with a median age of 7.5 years (range 1–16) all male. All patients but one patient with oligo JIA tested positive for ANA with immunofluorescence (IF). Intra-articular steroid injection (IASI) with triamcinolone hexacetonide (THA) was the first-line treatment for all patients in the cohort, given at the time of diagnosis, with a steroid dose of 1 mg/kg of knees, 0.5 mg/kg for other large joints and 0.3 mg/kg for small joints as accepted. Two of the 3 poly JIA patients were initially treated with systemic methotrexate (MTX) in addition to the intra-articular steroid injections. The median time from the time of disease diagnosis (i.e., first intra-articular steroid injection; sample 1) to the first follow-up after the intra-articular joint injection with steroids (sample 2) was 83 days (ranging 70–124). Further clinical and laboratory characteristics at the time of diagnosis and at the first follow-up after the steroid injections are described in Table 2, including the number of active joints, CRP, cJADAS10, NETs, NE, and MPO (Table 2).

**Table 1:** Patients demographics and characteristics

Patient	Sex (female/male)	Age at diagnosis (years)	Ethnicity Jewish Ashkenasi (1) Jewish Sefaradi (2)	Family history of autoimmune diseases (+ / −)	ANA IF status (+ / −)	Days btw diagnosis and IAIS	Days btw IAIS and follow-up	Treatment	Number of joints injected
Oligo 1	F	11	2	+	−	44	124	IASI	2
Oligo 2	F	3	1,2	+	+	6	70	IASI	1
Oligo 3	F	6	1,2	−	+	9	76	IASI	1
Oligo 4	F	3	1	−	+	5	70	IASI	1
Poly 1	M	2	1	+	+	18	83	IASI	5
Poly 2	M	14	2	+	+	6	84	IASI + MTX	8
Poly 3	F	3	1,2	−	+	0	85	IASI + MTX	4

*Oligo* Oligoarticular, *Poly* Polyarticular, *ANA IF* Anti-nuclear antibody immunofluorescent test, *IAIS* Intra-articular steroid injection, *MTX* Methotrexate

**Table 2:** Patient clinical characteristics

Patient	Number of active joints Dx	Number of active joints FTx	CRP (mg/l) Dx	CRP (mg/l) FTx	cJADAS10 Dx	cJADAS10 FTx	NETs (%) Dx	NETs (%) FTx	NE activity (mU) Dx	NE activity (mU) FTx	MPO activity (U) Dx	MPO activity (U) FTx
Oligo 1	2	0	0.06	0.14	14	3	70.5	34.3	8.3	6.6	1.17	1.36
Oligo 2	1	1	18.00	0.20	13	6	88.3	59.6	15.9	4.7	1.72	1.37
Oligo 3	1	0	1.62	0.03	3	0	^a^	27.5	3.3	4.9	0.80	0.29
Oligo 4	1	2	12.00	7.00	9	5	43.2	36.0	13.9	16.4	1.03	1.01
Poly 1	5	0	4.50	0.71	18	0	44.4	93.8	9.7	12.8	1.76	0.42
Poly 2	8	9	78.00	20.00	17	18	31.5	88.0	6.4	11.3	0.77	0.54
Poly 3	5	5	37.00	20.04	11	20	35.9	44.6	5.3	5.0	0.39	1.48

*Dx* at the time of diagnosis, *FTx* following treatment

^a^Not enough cells

### Correlation between NETs formation and disease activity score is statistically significant in oligo JIA patients

**Fig. 1:**
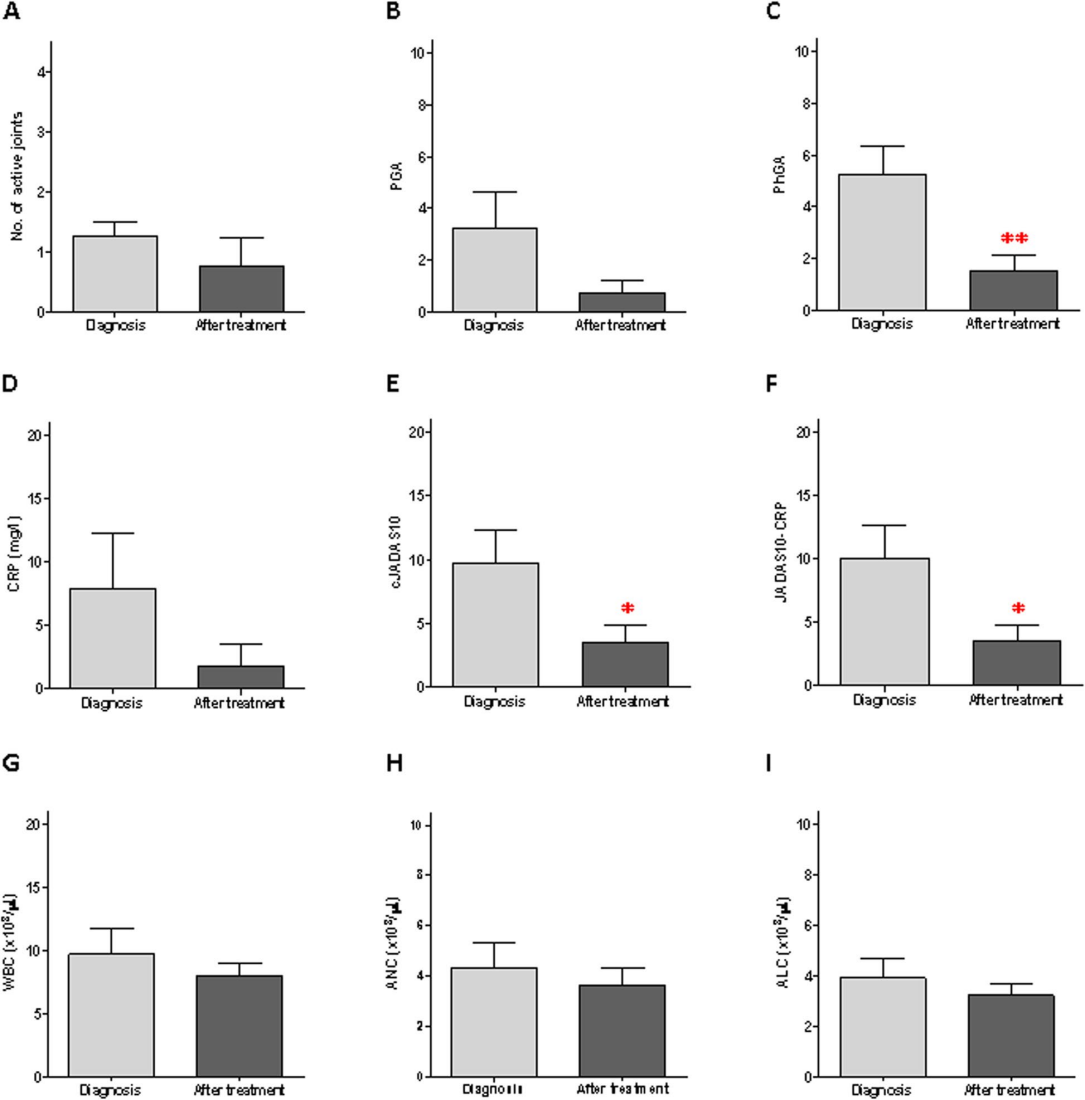
Oligoarticular JIA patient characteristics at diagnosis and first follow-up after treatment. Clinical and laboratory parameters and calculated disease activity scores at diagnosis and following steroid injection of 4 oligo JIA patients. **A** Number of active joints, **B** PGA, **C** PhGA, **D** CRP, **E** cJADAS10, **F** JADAS10-CRP; cell counts of** G** white blood cells, **H** neutrophils, **I** lymphocytes. PhGA, cJADAS10, and JADAS10-CRP significantly decreased after treatment compared to diagnosis (*P < 0.05, **P < 0.01)

**Fig. 2:**
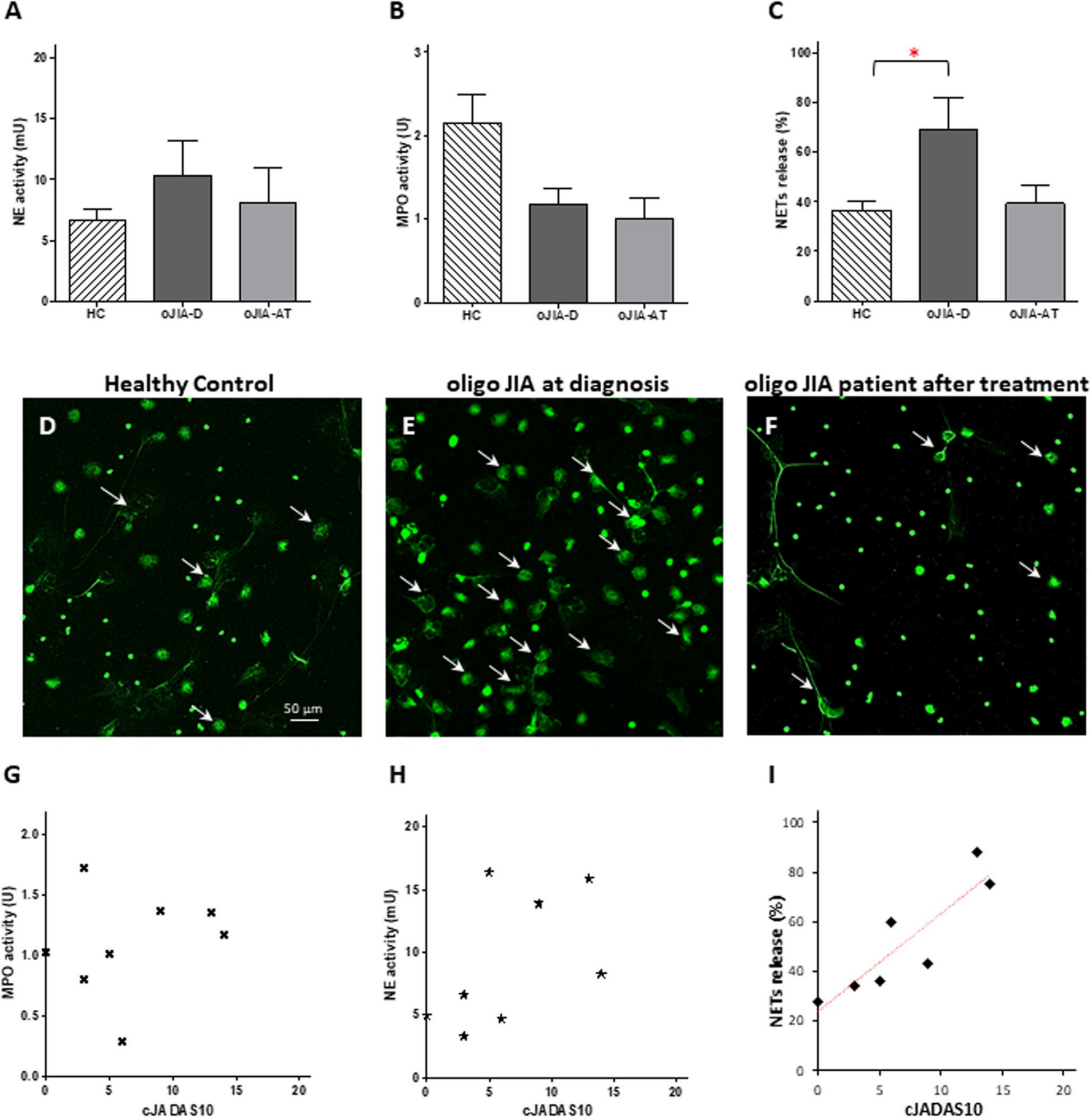
Neutrophil granular enzyme activities and NETs formation among oligo JIA patients.** A** NE activity, **B** MPO activity, and **C** percentage of NETs formation of oligo JIA patients (*N* = 4) compared to pediatric healthy controls (HC) (*N* = 6). NE activity and NETs were increased compared to healthy controls at diagnosis and decreased after anti-inflammatory treatment, only the difference between HC and oJIA at diagnosis was statistically significant. Representative immunofluorescent staining demonstrating NETs formation of **D** healthy control; **E** oligo JIA patient at diagnosis; **F** oligo JIA patient after anti-inflammatory treatment. Oligo JIA patients at diagnosis exhibit a significant increase in NETs formation in comparison to healthy control. Following anti-inflammatory treatment NETs formation in oligo JIA patients decreased. White arrows represent NETing neutrophils. **G** NE activity and **H** MPO activity showed no correlation with cJADAS. **I** NETs formation exhibited a statistically significant linear correlation with cJADAS10 (**P = 0.007)

In the oligo JIA patients, all the clinical and laboratory parameters decreased following intra-articular steroid injection (Fig. 1A–I). However, only PhGA, cJADAS-10, and JADAS-10 exhibited a statistically significant decline (Fig. 1C, E, F). NE activity (Fig. 2A) and NETs formation (Fig. 2C, D–F) were increased in oligo JIA patients at the time of diagnosis (oJIA-D) compared to the healthy controls (HC). However, only the elevation of NETs formation was statistically significant. Following intra-articular steroid injection, NE activity (Fig. 2A) and NETs formation (Fig. 2C) both showed a decrease. NE activity reduced from 10.4 mU ± 2.8 to 8.2 mU ± 2.8, while NETs formation declined from 68.9% ± 13.4% to 39.4% ± 7.0%, a comparable level to that of the healthy controls, whose NETs formation was 36.7% ± 3.7% (*P* = 0.72). Furthermore, NETs formation exhibited a statistically significant linear correlation with cJADAS-10 (*p* = 0.007) (Fig. 2I). There was no statistically significant correlation between NE, MPO activity, and cJADAS (Fig. 2G and H). Additionally, we calculated the correlation of all clinical and laboratory parameters with NETs formation and found a statistically significant correlation with PGA, PhGA, and JADAS10-CRP (Table 3).

**Table 3:** Correlation between NETs and clinical and laboratory parameters among oligoarticular JIA patients

Correlation		Correlation coefficient	*P* value	Significance
No. of active joints	NETosis	0.57	0.20	-
PGA	NETosis	0.95	0.003	**
PhGA	NETosis	0.94	0.007	**
CRP	NETosis	0.54	0.24	-
JADAS10-CRP	NETosis	0.93	0.007	**
WBC	NETosis	0.54	0.24	-
ANC	NETosis	0.72	0.40	-
ALC	NETosis	0.39	0.09	-

### NETs formation does not correlate with disease activity score in polyarticular JIA patients

**Fig. 3:**
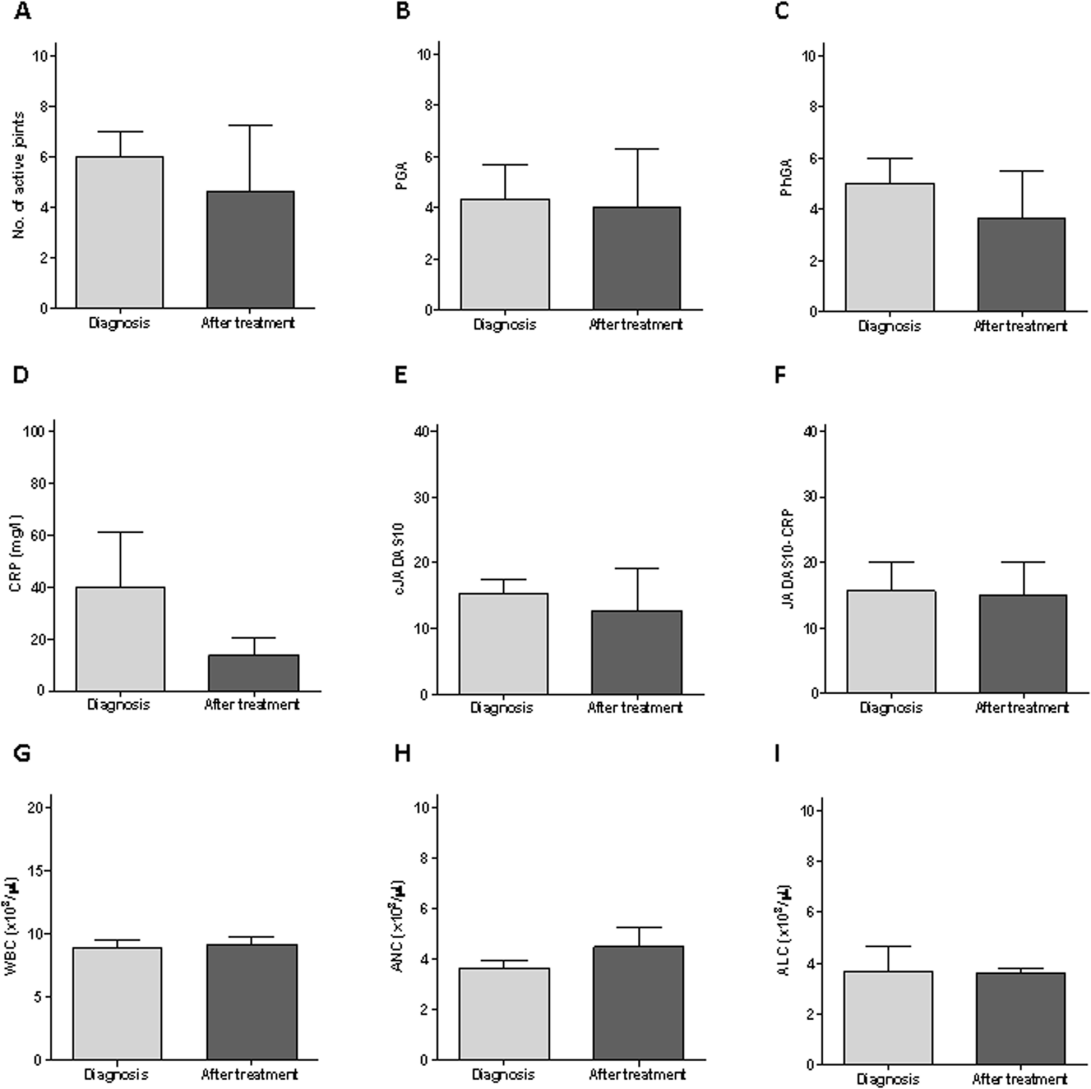
Polyarticular JIA patient characteristics at diagnosis and following treatment. Clinical and laboratory parameters and calculated disease activity scores at diagnosis and following steroid injection of 3 poly JIA patients. **A** Number of active joints, **B** PGA, **C** PhGA, **D** CRP, **E** cJADAS10, **F** JADAS10-CRP, cell counts of** G** white blood cells, **H** neutrophils, and **I** lymphocytes. None of the clinical and laboratory parameters changed significantly following treatment

**Fig. 4:**
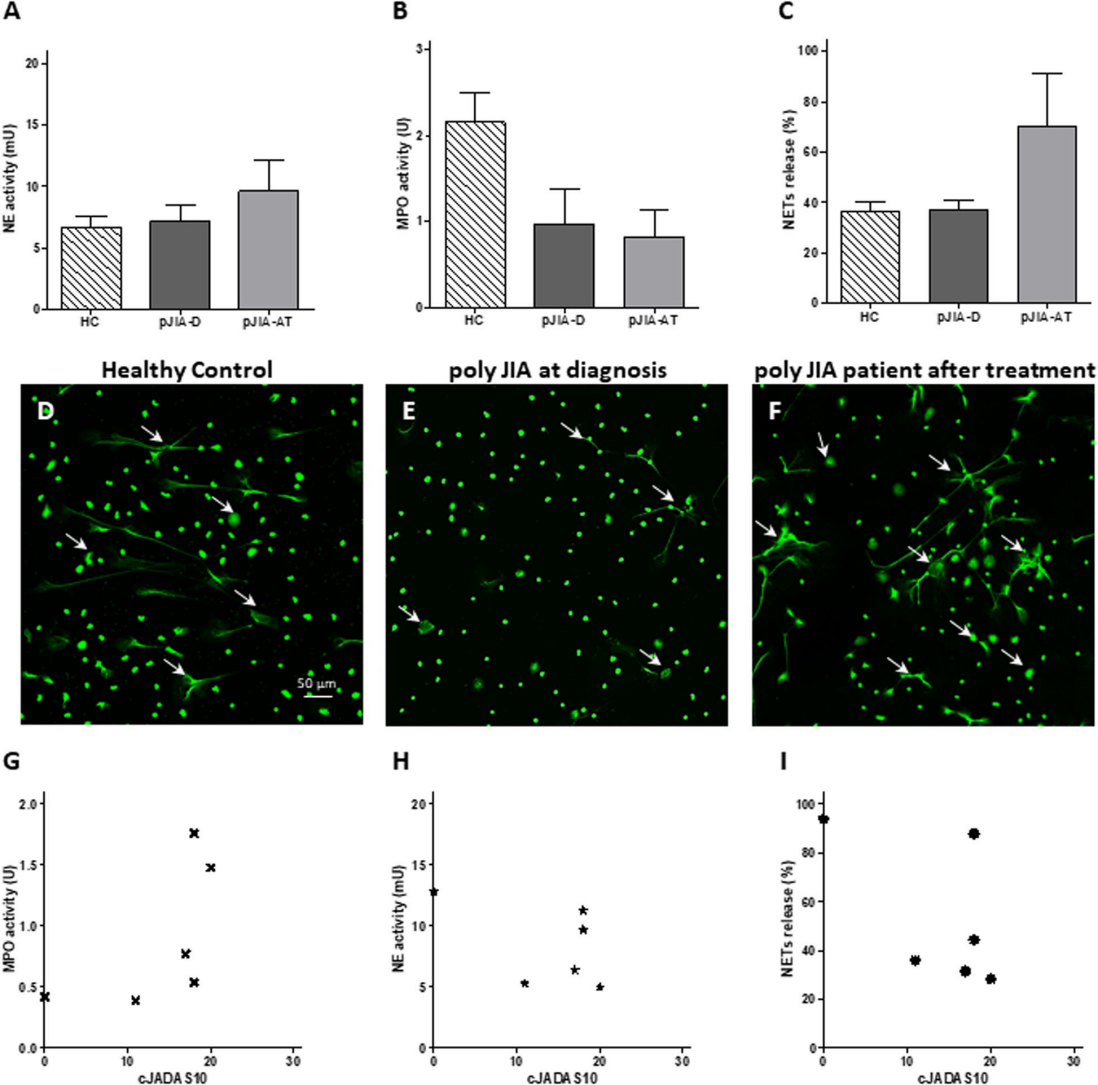
Neutrophil granular enzyme activities and NETs formation among poly JIA patients. **A** NE activity, **B** MPO activity, and **C** percentage of NETs formation of poly JIA patients (*N* = 3) compared to pediatric healthy controls (HC) (*N* = 6). NE activity and NETs were similar to HC at diagnosis and increased after anti-inflammatory treatment. MPO activity was decreased in JIA patients at diagnosis in comparison to HC and did not significantly change following treatment. All of the described parameters did not change significantly between the groups. Representative immunofluorescent staining demonstrating NETs formation of **D** healthy control; **E** poly JIA patient at diagnosis; and **F** poly JIA patient after anti-inflammatory treatment. Poly JIA patient at diagnosis exhibits similar NETs formation as healthy control. Following anti-inflammatory treatment, NETs formation in the poly JIA patient increased. White arrows represent NETing neutrophils. None of the parameters including **G** NE activity, **H** MPO activity, and **I** NETs formation exhibited a correlation with cJADAS

In the poly JIA patients, all the clinical parameters including the number of active joints, PGA, PhGA, and CRP values showed a minor, non-significant decrease following anti-inflammatory treatment initiation (Fig. 3A–F). White blood cell counts did not change (Fig. 3G–I). NE activity (Fig. 4A) and NETs formation were measured at the same level in poly JIA patients as in the healthy controls at the time of diagnosis and have increased after treatment (Fig. 4C, D–F). MPO activity showed a lower level compared to healthy controls at diagnosis and has mildly reduced after treatment (Fig. 4B). Neither of the neutrophil-related parameters exhibited a correlation with clinical disease activity score cJADAS-10 (Fig. 4G–I). Furthermore, none of the other clinical characteristics showed a correlation with NETs formation (Table 4).

**Table 4:** Correlation between NETs and clinical and laboratory parameters among polyarticular JIA patients

Correlation		Correlation coefficient	*P* value	Significance
No. of active joints	NETosis	− 0.21	0.72	-
PGA	NETosis	− 0.52	0.30	-
PhGA	NETosis	− 0.70	0.17	-
CRP	NETosis	− 0.77	0.10	-
JADAS10-CRP	NETosis	− 0.14	0.80	-
WBC	NETosis	0.41	0.42	-
ANC	NETosis	− 0.26	0.66	-
ALC	NETosis	0.43	0.42	-

### Influence of clinical characteristics on NETs formation and NE activity

**Fig. 5:**
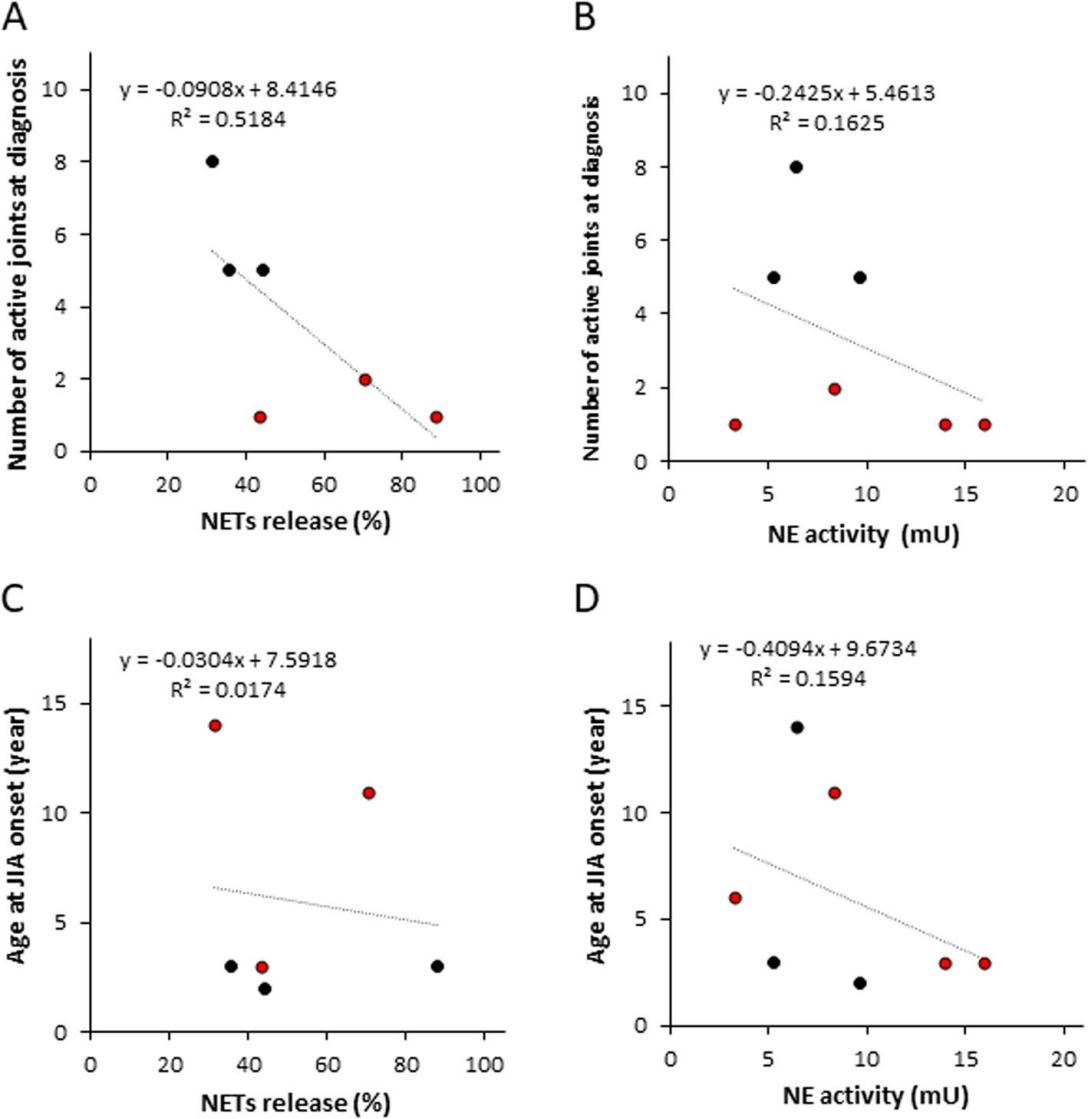
Linear regression analysis of NETs release and NE activity with clinical characteristics of JIA patients. Red circles represent oligo JIA patients; black circles represent poly JIA patients on the graphs. Low evidence of correlation was shown with all clinical characteristics analyzed. Changes in NETs release and NE activity might be influenced by the number of clinical characteristics like the number of active joints (**A**, **B**) and age at JIA onset (**C**, **D**)

We performed further analysis on the entire cohort at diagnosis as one group to gain more information concerning the influencing factors on NETs and NE activity and to evaluate the feasibility as biomarkers. Due to the sample size of the patient population, which is a limiting factor, a bivariate analysis in the form of linear regression was performed between NETs formation and NE activity and clinical characteristics including sex, age at JIA onset, ANA positivity, and number of active joints (Table 5 and Fig. 5). No significant correlation was shown, likely due to the low sample size. The best correlations were found between NETs release and the number of active joints with *R*^2^ = 0.518 and *P* = 0.11 (Fig. 5A) as well as NE activity with the age at JIA onset and number of active joints (*R*^2^ = 0.159 and 0.163 respectively and *P* = 0.37 in both cases) (Fig. 5B, D).

**Table 5:** Linear regression analysis between NETs release, NE activity, and clinical characteristics of JIA patients

Linear regression	NETs release		NE activity	
	***R***^**2**^	***P***	***R***^**2**^	***P***
Sex	0.250	0.31	0.019	0.77
Age at onset (years)	0.017	0.80	0.159	0.37
ANA positivity	0.161	0.43	0.004	0.89
No. of active joints	0.518	0.11	0.163	0.37

## Discussion

In the last decade, neutrophils and NETs formation have received a lot of attention as their role in non-infectious conditions was revealed, including in the pathogenesis of autoimmune and inflammatory diseases [9]. JIA has long been established as an autoimmune disease, triggered by aberrant recognition of autoantigens by T-cells. However, system biology approaches investigating JIA have proposed the complex interaction between innate and adaptive immunity [27]. Moreover, recent data suggest an important role for neutrophils in JIA pathogenesis [28, 29].

In this prospective inception cohort, we explored NETs formation and the enzymatic activity of neutrophil granular enzymes NE and MPO in children with oligoarticular and polyarticular JIA in addition to standard clinical parameters including white blood cell counts, CRP levels, and disease activity scores. We also addressed the correlation of JIA disease activity scores (JADAS) with NETs formation and NE and MPO activity as potential biomarkers for JIA.

In our pilot study, we found that oligo JIA patients showed significantly increased NETs compared to healthy controls at the time of diagnosis. Moreover, neutrophil function, specifically NETs formation, correlated with the disease activity following the steroid injection. Following the treatment with intra-articular steroid injection, NETs formation showed a decreasing trend in comparison to samples at diagnosis, though not significant likely due to the small sample size (*N* (oligo JIA) = 4 and *N* (control) = 6). In the case of poly JIA patients, the NETs at time of diagnosis were not necessarily high, and no correlation was found between neutrophil function and disease activity. These differences may again relate to the small sample size of the cohort, as there is little data in the literature to support the difference that we demonstrated in the neutrophil parameters between oligo and poly JIA. However, it is possible that the pathogenesis of the two is different.

There is limited data in the literature on the role of neutrophils and their function in oligo and poly JIA. A recent study reported the presence of both phenotypically and functionally altered neutrophils in the inflamed joint in oligoarticular JIA [28]. Neutrophils in the synovial fluid were activated, had an aged phenotype, had gained monocyte-like features, and had impaired phagocytic capacity. The impairment in phagocytosis and oxidative burst was associated with the phenotype shift. Another study showed that neutrophils from synovial fluid of JIA patients display a hyperactivated phenotype [29]. Overall, the data from the literature supports neutrophil activation during the active state of JIA, though how treatments affect neutrophil function is not conclusive.

Once treating children with JIA, frequent and timely assessment of their clinical response to therapy and disease activity is crucial in a goal to receive clinically inactive disease or low disease activity. The main assessment currently used in JIA, the composite disease activity score, was developed in 2009 and was named Juvenile Arthritis Disease Activity Score (JADAS) [30]. Because inflammatory markers are not always obtained or available at a visit, and their efficacy is often debated, a three-variable version of the JADAS that does not include the acute-phase reactant, termed the clinical JADAS (cJADAS), has been proposed [31]. Though these markers help to follow up disease progression and evaluate treatment effectiveness, the components of PGA and PhGA are rather subjective. There are attempts to develop more specific laboratory biomarkers, such as serum proteins, the neutrophil activation marker S100A12, and the phagocyte activation marker myeloid-related proteins 8 and 14 heterocomplexes (MRP8/14), which were found to be more specific in predicting response to methotrexate and JIA flare than other clinical and laboratory data [32, 33]. It was also revealed that neutrophils in children with JIA are primed to release granular proteins without relation to medical treatment, whereas signs of increased turnover and sequestration of neutrophils are reduced by treatment [34]. However, none of these laboratory markers was implemented in common clinical practice and, therefore, there is a need for the search of putative biomarkers for timely assessment of JIA. In this respect, the finding that NETs formation declines after treatment, coupled with a decreased cJADAS-10 score in oligo JIA might point to a potential biomarker for disease activity.

There are several limitations to our study. Most notably, the size of the cohort is smaller than expected, mainly due to the exclusion of many patients who had no sample from the time of diagnosis. In addition, the method used for neutrophil isolation required fresh blood, which has limited the study to be a single-center study. However, this is a pilot report and we are aiming to increase the cohort for our future reports. In addition, blood samples were used for the study but we did not examine synovial samples, which would potentially provide another perspective to the results.

## Conclusions

In conclusion, this is the first study exploring the link between NETs formation and JIA activity. We demonstrated a statistically significant linear correlation between NETs formation and cJADAS-10 in oligo but not in poly JIA patients. Hence, we suggest that NETs formation might play a role in JIA activity and may serve as a putative biomarker. This is a pilot study and more data and in-depth understanding is needed to validate these initial results.

## Data Availability

The authors confirm that the data supporting the findings of this study are available within the article. The raw data was generated in the Tel Aviv Sourasky Medical Center and is available on request from the corresponding author.

## Abbreviations

JIA: Juvenile idiopathic arthritis
Oligo: Oligoarticular
Poly: Polyarticular
ANA: Anti-nuclear antibody
RF: Rheumatoid factor
NETs: Neutrophil extracellular traps
NE: Neutrophil elastase
MPO: Myeloperoxidase
SLE: Systemic lupus erythematosus
RA: Rheumatoid arthritis
IBD: Inflammatory bowel disease
SF: Synovial fluid
ILAR: International League of Associations for Rheumatology
PhGA: Physician’s global assessment
VAS: Visual analog scale
PGA: Parent/patient-reported global assessment
WBC: White blood cells
ANC: Absolute neutrophilic count
ALC: Absolute lymphocytic count
CRP: C-reactive protein
IFA: Indirect immunofluorescence assay
Anti-CCP: Anti-cyclic citrullinated peptide
JADAS-10: Juvenile Activity Disease Activity Score-10
PBS: Phosphate buffered saline
EDTA: Ethylenediamine tetra-acetic acid
BSA: Bovine serum albumin
IASI: Intra-articular steroid injection
THA: Triamcinolone hexacetonide
MTX: Methotrexate
